# Lumbar Arachnoid Cyst Presenting as Arachnoiditis on MRI: A Case Report and Diagnostic Approach

**DOI:** 10.7759/cureus.96205

**Published:** 2025-11-06

**Authors:** Alex A Dos Santos, Anuksha Varghese, Ammar Al-Amour, Mansour Afshani, Nora Nikprelevic, Parker Mckethan

**Affiliations:** 1 Radiology, American University of the Caribbean, Miami, USA; 2 Medicine, Ross University School of Medicine, Miami, USA; 3 Medicine, Jordan University of Science and Technology, Irbid, USA; 4 Neurology, Cleveland Clinic Florida, Weston, USA

**Keywords:** arachnoiditis, ct myelogram, empty thecal sac, lumbar arachnoid cyst, lumbar spine, neurological diagnosis

## Abstract

Lumbar arachnoid cysts (ACs) and arachnoiditis may appear similar on magnetic resonance imaging (MRI) and may appear difficult to diagnose accurately. Considering that lumbar arachnoid cysts have a benign source, in contrast to arachnoiditis, surgical intervention poses a challenge. We present a case initially assumed to be arachnoiditis but later confirmed as an arachnoid cyst by MRI and computed tomography (CT) myelogram. The goal of this report was to provide a clearer understanding of the pathophysiological differences and key diagnostic features, including imaging techniques and clinical presentations that distinguish these two conditions.

A 46-year-old Caucasian female presented to the emergency department with complaints of back pain and lower-extremity weakness with associated saddle paresthesia. Clinical examination revealed hyporeflexia with a diminished sensation in the left lower extremity. Accurate diagnosis using MRI and a comprehensive clinical approach is vital for early recognition and differentiation of cauda equina syndrome, spinal stenosis, arachnoiditis, spinal abscess, and epidural hematoma. In this case, the initial lumbar MRI demonstrated an empty thecal sac at L4-L5, which can be seen in cases of arachnoiditis and inflammatory arthropathy. With a negative inflammatory lumbar puncture and unresolved symptoms post methylprednisolone treatment, CT myelogram was ordered, which confirmed the presence of a lumbar arachnoid cyst at L4-L5. While arachnoiditis is primarily managed with symptomatic treatment, this patient had a spinal arachnoid cyst, which required surgical intervention to relieve compression and symptoms.

## Introduction

Arachnoid cysts and arachnoiditis are two distinct, although sometimes related, conditions that affect the arachnoid membrane, which is one of the three protective layers surrounding the brain and spinal cord. Depending on the location, size, and degree of compression of the adjacent structures, these conditions can lead to a range of neurological symptoms. Although both are rare, they can have significant clinical implications in affected individuals.

Arachnoid cysts are fluid-filled sacs that develop between the brain or spinal cord and the arachnoid membrane. These cysts are typically congenital, but can also be acquired due to trauma, infection, or other causes [1,2]. Most arachnoid cysts are asymptomatic and are often discovered incidentally during imaging studies performed for other reasons [3-5]. However, when symptomatic, they can cause a variety of neurological symptoms, including headaches, seizures, back pain, sensory disturbances, and motor deficits. Symptoms depend on cyst size and location, with larger cysts being more likely to exert pressure on the surrounding brain or spinal cord structures, leading to clinical manifestations [3].

Spinal arachnoid cysts account for only 1-3% of cases, with the remainder being intracranial [4]. Although uncommon, spinal arachnoid cysts may result in significant neurological impairment secondary to compression of the spinal cord or nerve roots. The clinical presentation varies depending on the cyst’s size and location, and may include monoparesis, radicular pain, spastic quadriparesis, sensory disturbances, or sphincter dysfunction such as incontinence and neurogenic bladder [5]. The majority of arachnoid cysts are situated dorsal to the spinal cord, with an estimated distribution of 80% in the thoracic region, 15% in the cervical region, and 5% in the lumbar region [3]. Diagnosis is usually made through advanced imaging techniques, such as magnetic resonance imaging (MRI), which helps to determine cyst size, location, and any potential complications, including compression of adjacent neural structures [1].

Arachnoiditis is an inflammatory condition of the spinal arachnoid membrane that can result in scarring, fibrosis, and disruption of the normal cerebrospinal fluid flow [3,5]. It can be caused by various factors, including infection, trauma, spinal surgery, or the introduction of foreign substances into the spinal canal (such as contrast agents or medications) [6,7]. Although the pathophysiology of arachnoiditis is complex, it generally involves irritation and inflammation of the arachnoid membrane, which can disrupt the normal flow of cerebrospinal fluid and cause spinal cord or nerve root compression [6]. Chronic inflammation may lead to the formation of adhesions and scar tissue, which can cause nerve root compression, pain, sensory disturbances, and motor deficits [5,6]. Arachnoiditis is often associated with severe, persistent pain, which may be localized or radiated to other areas depending on the affected region of the spine. Symptoms of arachnoiditis may include lower back pain, radicular pain, numbness, tingling, and muscle weakness, often exacerbated by certain movements or postures.

Both arachnoid cysts and arachnoiditis are important conditions to be recognized in patients presenting with neurological symptoms, particularly pain, sensory changes, and weakness. Given that these conditions may be similar to other spinal pathologies, thorough clinical evaluation and imaging workups are essential for accurate diagnosis and appropriate management. While arachnoid cysts can often be managed conservatively, larger cysts or those causing significant compression may require surgical intervention [1,4]. Arachnoiditis, however, is primarily managed through symptom control with no definitive cure, although surgical or interventional treatment may occasionally be considered in severe cases [8].

Understanding the pathophysiology, clinical presentation, and diagnostic strategies for arachnoid cysts and arachnoiditis is crucial for effective treatment and for improving the quality of life of affected individuals. This case emphasizes the diagnostic challenge posed by a rare condition. In a unique presentation, a lumbar arachnoid cyst mimicked arachnoiditis, highlighting the need for multimodal evaluation to clarify overlapping features and ensure proper patient management.

## Case presentation

A 46-year-old Caucasian female with a history of Crohn’s disease, recent right colectomy, and sacroiliitis presented to the emergency department (ED) with two days of lower back pain and numbness/tingling in the bilateral lower extremities. Ambulation was also associated with severe pain. The patient’s back pain had been chronic since her teens, with a history of scoliosis and sciatica. This was confirmed by a recent CT scan, which revealed bilateral sacroiliitis.

**Figure 1:**
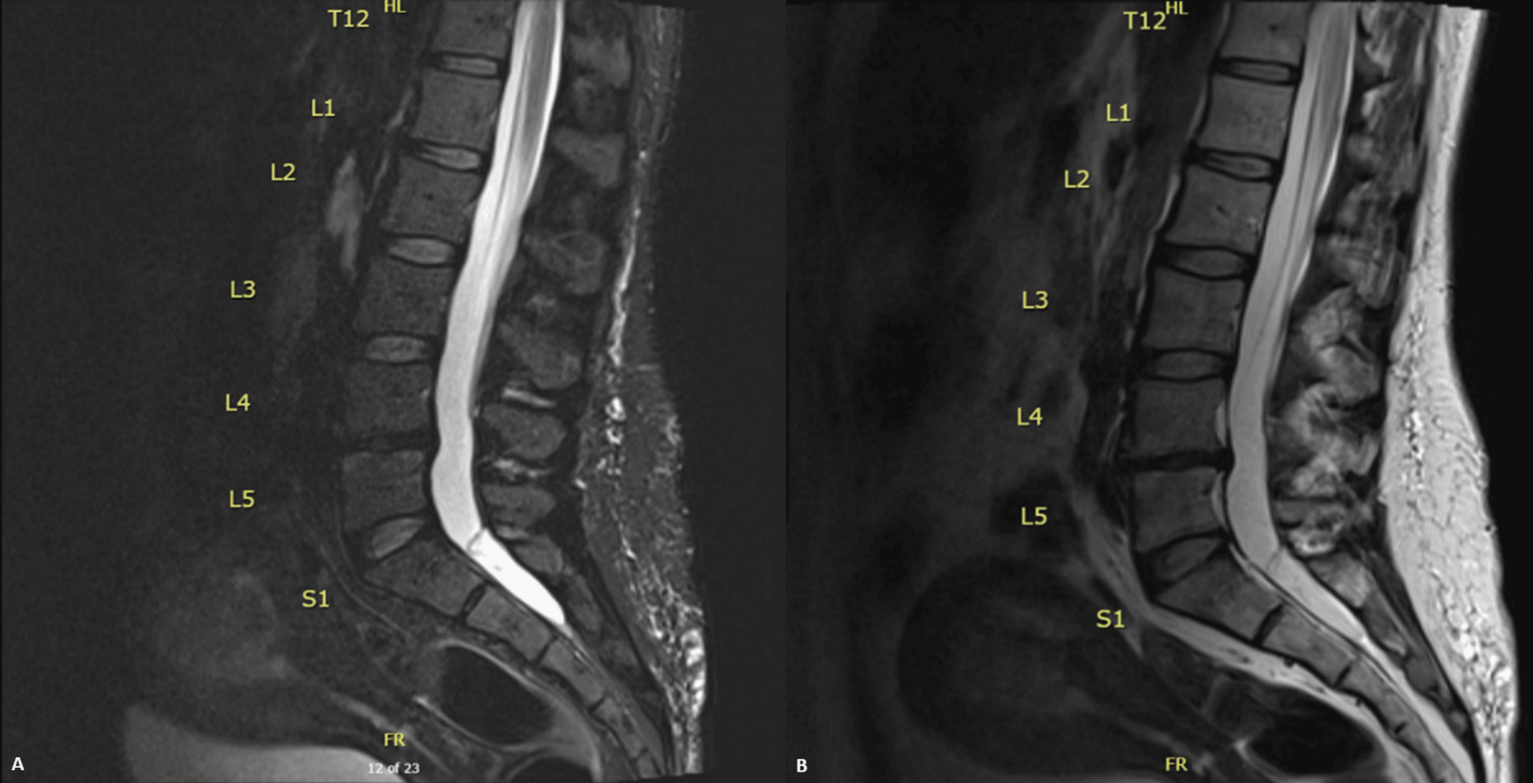
Initial MRI lumbar spine sagittal view. From left to right, image A shows initial findings of the patient from a lumbar MRI short tau inversion recovery sequence (STIR) of degenerative disc changes at L4-L5 and peripherally located nerve roots, and image B shows the T2 sequence.

**Figure 2:**
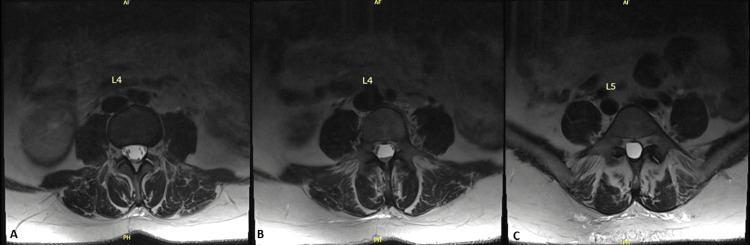
Initial MRI transverse view. From left to right, these images show the initial T2 MRI findings, which reveal peripheral displacement of the nerve roots. Image A is the superior portion of the 4th lumbar vertebra. Image B is the inferior portion of the 4th lumbar vertebra. Image C is the 5th lumbar vertebra. The transition of the images demonstrates an "empty thecal sac" appearance, which can be seen in patients with inflammatory arthropathy as well as arachnoiditis.

In the ED, neurological examination revealed urinary incontinence, saddle anesthesia, and decreased rectal tone, suggesting cauda equina syndrome. Neurological examination revealed reduced strength in hip flexion, knee extension, knee flexion, plantar flexion, and bilateral hyporeflexia in the lower extremities. The patient underwent an MRI of the lumbar spine, which did not show any cord compression but revealed an empty thecal sac at L4-L5, a finding that can be seen in patients with inflammatory arthropathy or arachnoiditis (Figures 1A, 1B, 2A-2C). The neurosurgeon indicated that she did not require surgical intervention and was admitted to the hospital for symptom management.

During her stay, an MRI of the brain and cervicothoracic spine did not reveal any acute abnormalities related to her symptoms. A lumbar puncture was performed at L3-L4 for further work-up of potential infectious and inflammatory causes of symptoms, revealing a total nucleated cell count of 3, cerebrospinal fluid (CSF) protein of 52 mg/dL, CSF glucose of 79 mg/dL, and a negative BioFire test result. With a suspicion of an inflammatory cause for the patient’s symptoms, intravenous methylprednisolone was started.

**Figure 3:**
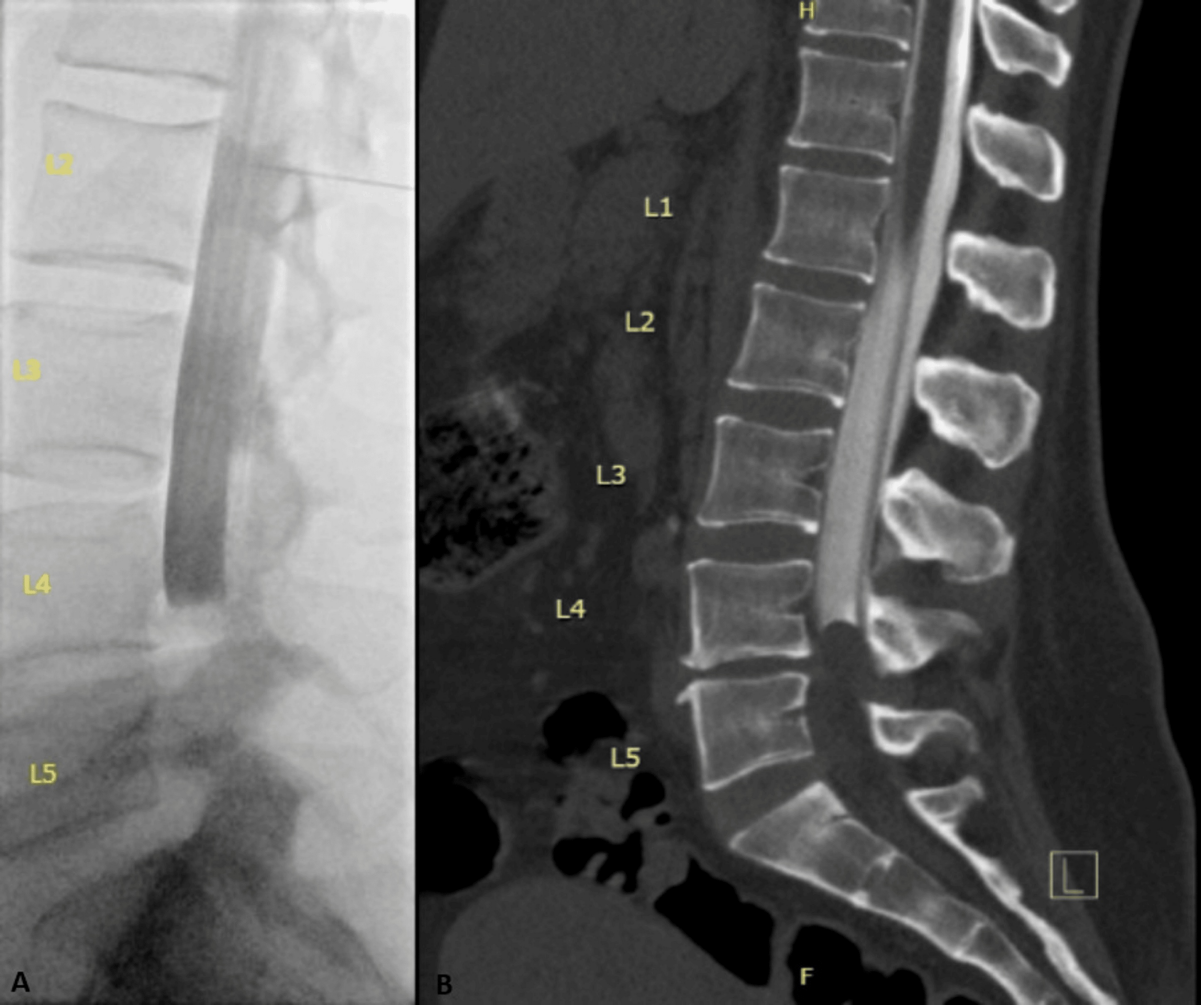
Confirmation of arachnoid cyst beginning at L4. From left to right, these images confirm the presence of an arachnoid cyst. Image A is an X-ray myelogram showing intrathecal injection of contrast material for a CT lumbar myelogram. Image B is a CT lumbar myelogram demonstrating complete attenuation of contrast enhancement below the mid-4th lumbar vertebra.

Despite treatment, the patient continued to complain of pain and reported no symptom alleviation. A revisited review of the lumbar spine MRI revealed an unusual appearance of an empty thecal sac, namely, the appearance of a mass effect on the nerves surrounding the periphery of the spinal cord. Computed tomography (CT) myelography showed complete attenuation of contrast-related enhancement below the mid-L4 level, correlating with the region of abnormal cauda equina nerve root displacement and empty thecal sac appearance on recent MRI of the lumbar spine (Figures 3A, 3B). These findings suggested an arachnoid cyst causing complete effacement of the spinal canal at the L4-L5 and L5-S1 levels.

**Figure 4:**
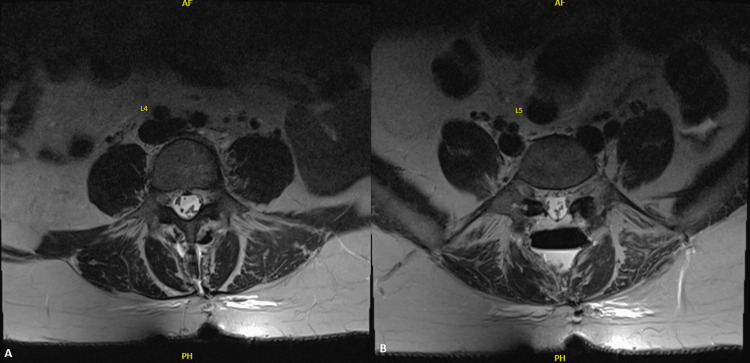
Postoperative T2 MRI transverse view. From left to right, these images demonstrate the changes seen after laminectomies and partial medial facetectomies at L4-L5. Image A is the 4th lumbar vertebra. Image B is the 5th lumbar vertebra. The nerve roots are now seen within the spinal canal without peripheral displacement.

The neurosurgeon performed a decompressive laminectomy, medial facetectomy, foraminotomy, and drained the arachnoid cyst to the subarachnoid space via a cyst fenestration and wall excision. The patient reported immediate relief of symptoms after surgery and showed immediate signs of recovery. A repeat MRI of the lumbar spine revealed interval fenestration/resection of the previous arachnoid cyst within the lower lumbar canal and resolution of the previous mass effect on the cauda equina nerve roots, which were no longer peripherally displaced (Figures 4A, 4B).

## Discussion

The majority of arachnoid cysts are believed to arise secondary to a congenital defect in the diverticulum of the dura, whereas non-idiopathic etiologies include trauma, prior surgery, spondylosis, or chronic arachnoiditis [9]. In our patient, with chronic symptoms, history of inflammatory disease, absence of spinal trauma, and empty thecal sac sign on MRI, establishing the correct diagnosis was particularly challenging.

To the best of our knowledge, reports of a congenital intradural lumbar arachnoid cyst mimicking arachnoiditis are scarce in existing literature. When discussing a lumbar arachnoid cyst in middle-aged patients, it is more commonly associated with a history of epidural injections [9,10]. These patients typically present with similar symptoms such as back pain, lower limb weakness and pain, and sphincteric dysfunction [9,10].

**Figure 5:**
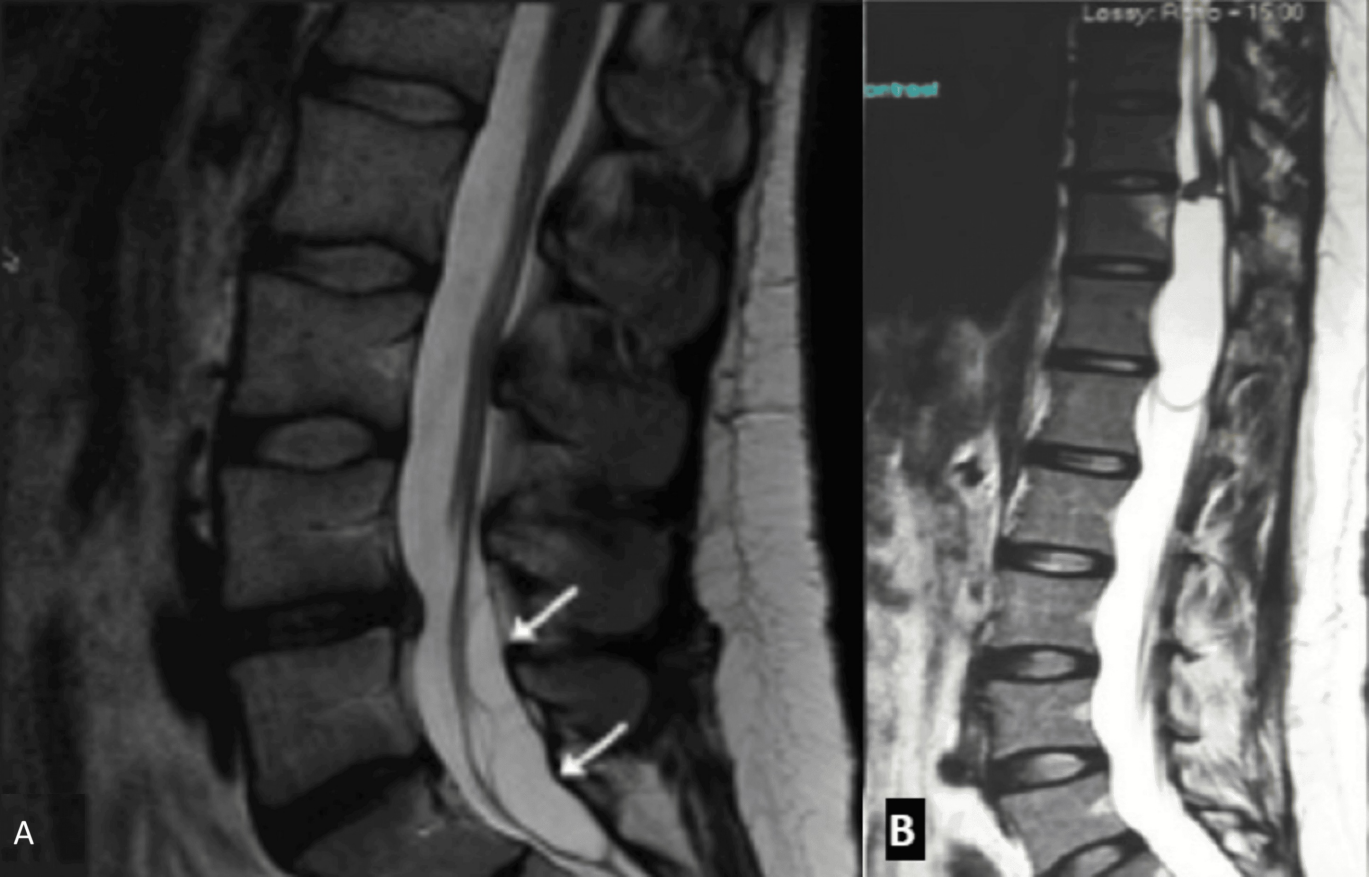
Post-epidural spinal arachnoid cyst. Image A is a sagittal axial T2-weighted MRI of the lumbrosacral spine showing two well-defined, loculated, intradural arachnoid cysts (arrows) in a 36-year-old female patient [9]. Image B is a sagittal axial T2-weighted MRI of the lumbrosacral spine showing an intradural extramedullary cyst in spinal levels T11 through L1 in a 43-year-old female patient [10].

**Figure 6:**
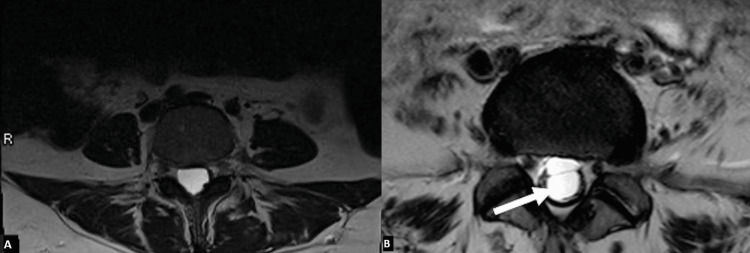
Empty thecal sac sign. Image A is an axial MRI of the spine without contrast, revealing adherent cauda equina nerve roots to the thecal sac, creating an "empty thecal sac" sign in a 33-year-old female patient [12]. Image B is an axial MRI of the lumbrosacral spine examination revealing the distorted nerve roots, adherent to the thecal sac, creating the “empty thecal sac” sign (arrow) typical for adhesive arachnoiditis in a 56-year-old female patient [13].

We believe that the diagnostic challenge in this case stemmed primarily from the atypical imaging characteristics of the cyst. In most reported cases, arachnoid cysts are more easily recognized by their well-defined borders, particularly in the sagittal view (Figures 5A, 5B) [3]. In contrast, the arachnoid cyst in our case filled the entire spinal canal, causing clumping and peripheral displacement of the nerve roots. This pattern produced the characteristic appearance of an “empty thecal sac” sign, which is classically associated with adhesive arachnoiditis (Figures 6A, 6B) [11]. Notably, adhesive arachnoiditis can also present with similar clinical features to a lumbar arachnoid cyst [12,13].

In our case, initial MRI findings suggested arachnoiditis given the seeming appearance of an empty thecal sac. However, the uniform displacement of the nerves to the periphery of the spinal canal with flat borders towards the center and the absence of inflammatory changes raised the suspicion of a different diagnosis. A CT myelogram ultimately confirmed the presence of an arachnoid cyst, enabling appropriate and targeted intervention. This distinction was critical, as management for arachnoiditis would not have addressed the underlying pathology and could have prolonged the patient’s symptoms without relief.

## Conclusions

This case highlights the diagnostic challenges associated with a spinal arachnoid cyst versus arachnoiditis and other differential diagnoses. MRI findings in a 46-year-old female revealed an arachnoid cyst that initially presented with clinical features resembling arachnoiditis. Ultimately, a CT myelogram and lumbar MRI clarified the diagnosis of an arachnoid cyst, underscoring the importance of maintaining a broad differential diagnosis for spinal nerve dysfunction and the importance of reviewing imaging to rule in/out differential diagnosis. Thorough clinical evaluation, combined with advanced imaging techniques, is essential for accurately diagnosing and differentiating these conditions to guide appropriate management. Early recognition and intervention are critical for reducing the risk of long-term neurological complications.
